# Gaucher disease in Brazil: a comprehensive 16 year retrospective study on survival, cost, and treatment insights

**DOI:** 10.3389/fphar.2024.1433970

**Published:** 2024-09-26

**Authors:** Marcus Carvalho Borin, Juliana Alvares-Teodoro, Francisco Assis Acurcio, Augusto Afonso Guerra

**Affiliations:** ^1^ Department of Social Pharmacy, Faculty of Pharmacy, Federal University of Minas Gerais, Belo Horizonte, Brazil; ^2^ SUS Collaborating Center for Technology Assessment and Excellence in Health, Faculty of Pharmacy, Federal University of Minas Gerais, Belo Horizonte, Brazil

**Keywords:** gaucher disease (GD), real-world data (RWD), enzyme replacement therapy, substrate synthesis inhibitor, survival analysis, cohort study, record linkage, Brazil

## Abstract

**Background:**

Gaucher’s disease (GD), a lysosomal storage disorder, poses significant treatment challenges. This 23-year study assesses survival rates and treatment efficacy in Brazilian GD patients, integrating data from a 16-year cohort (2000–2015) and the TABNET/DATASUS medicines distribution data (1999–2022).

**Objective:**

To investigate the survival of GD patients in Brazil, identifying key risk factors and evaluating the impact of treatments funded by the Brazilian National Health System (SUS).

**Methodology:**

A 16-year retrospective cohort study was conducted using the National Database of SUS. Patients diagnosed with GD and treated with Enzyme Replacement Therapy (ERT) or Substrate Synthesis Inhibition (SSI) from 2000 to 2015 were included. Survival analysis was performed using Kaplan-Meier method and Cox proportional hazards model. The data from TABNET/DATASUS system from 1999 to 2022 was used to assess the trend in drug distribution beyond the main cohort.

**Results:**

The study included 1,234 patients. Survival rates at 5 and 10 years were 93.2% and 88.5%, respectively, with age and comorbidities like diabetes, cardiovascular diseases, and Parkinson’s disease significantly affecting survival. Patients who received doses lower than DDD (n = 880) demonstrated a survival probability of 91.8%. In contrast, those with doses equal to the DDD (n = 15) showed a 100% survival probability, as no events were observed in this group. The greater than DDD group (n = 339) exhibited a survival probability of 81%. A log-rank test indicated a borderline statistical significance (*p* = 0.058) in the survival distributions among the different DDD adherence, with the lower dose group showing a favorable trend.

**Conclusion:**

This study provides insights into the survival rates and associated risk factors for GD patients in Brazil, contributing to the global understanding of GD and its management. While we acknowledge the inherent limitations of relying largely on electronic medical records and categorical codes, our findings underscore the need for early diagnosis, timely initiation of treatment, effective management of comorbidities, and personalized dosing strategies to improve patient outcomes. Future studies should aim to incorporate clinical verification of electronic data to further enhance the reliability and applicability of these findings.

## 1 Introduction

Gaucher’s disease (GD) is an autosomal recessive lysosomal storage disorder caused by a deficiency of the enzyme glucocerebrosidase. This deficiency leads to the accumulation of its primary substrate, glucocerebroside, in the lysosomes of macrophages, especially in the spleen, liver, and bone marrow. In severe cases, it can affect the lungs, kidneys, and central nervous system (Cox et al., 2015; Grabowski, 2005; Grabowski et al., 1995).

GD is pan-ethnic, but with a high incidence in the Ashkenazi Jewish population, reaching 1:855 live births, compared to just 1:57,000 in the general population. In the USA, the estimated incidence in descendants of Jews is 1:400–1,000 newborns, while in the general population, it is only 1:40,000–60,000. Some studies estimate an incidence in the general population of 1:75,000 newborns (Futerman and Zimran, 2006; Grabowski, 2005; Sobreira and Bruniera, 2008).

The disease manifests in three main types, distinguished primarily by the presence and severity of neurological involvement. Type I GD, the most common and non-neuronopathic form, varies greatly in symptoms and severity, often presenting in adulthood. Type II, the rarest acute neuronopathic form, is marked by severe neurological changes and is often fatal in early childhood. Type III GD, a subacute neuronopathic form, combines features of the first two types and presents with gradually progressive neurological dysfunction (Berrebi, Wishnitzer, and Von-der-Walde, 1984; Sidransky, Sherer, and Ginns, 1992).

Historically, treatment for GD was limited to palliative care, with splenectomy being a common intervention. However, advancements have led to two main treatment modalities: Enzyme Replacement Therapy (ERT) and Substrate Synthesis Inhibition (SSI) (Cassinerio, Graziadei, and Poggiali, 2014).

ERT involves intravenous administration of a recombinant form of the glucocerebrosidase (GCase) enzyme, with dosage varying according to each patient’s clinical manifestations. Typically, infusions occur biweekly and require supervision by a trained healthcare professional. The goal of ERT is to replenish deficient GCase in GD patients, reducing glucocerebroside (GC) substrate accumulation and improving symptoms. ERT began with the use of human placenta-purified GCase enzyme, Alglucerase, later replaced by the recombinant form (imiglucerase). ERT forms approved by the Food and Drugs Administration (FDA) and National Health Surveillance Agency (Anvisa) in Brazil and commercially available for GD treatment are: imiglucerase, alfavelaglicerase, and alfataliglicerase. In addition to the three enzyme replacement therapies (ERTs) approved in the US, generic imiglucerase has been used in some countries, though its clinical efficacy and safety have not been studied as extensively. The differences among these forms lie in their production methods and compositions. However, ERT has disadvantages, including high cost (up to $300,000 per patient per year), inability of the enzyme to cross the blood-brain barrier (more effective for GD type I), biweekly intravenous administration, non-uniform efficacy and enzyme distribution across different tissues, and increased production of antibodies against the recombinant enzyme. ERT has been highly effective in reversing visceral, hematological, and bone manifestations, showing a 50%–60% reduction in spleen volume and a 30%–40% reduction in liver size within two to 5 years of treatment, as well as a doubling of platelet count within 5 years of ERT (Mistry et al., 2017).

SSI acts directly on the accumulated substrate and consists of small compounds that inhibit GC substrate synthesis, diffusing rapidly into various tissues, including bones and the CNS. Instead of replenishing the deficient enzyme, this approach inhibits GC accumulation inside lysosomes by inhibiting glycosylceramide synthase, responsible for GC synthesis. FDA and Anvisa-approved inhibitors commercially available for GD treatment are miglustat and eliglustat. It is important to differentiate between miglustat and eliglustat. Eliglustat, approved in Brazil after our data collection period, has been shown to be effective as a first-line treatment for Gaucher disease type 1, with a different side effect profile compared to miglustat. The medication is administered orally daily. This approach’s advantage is the use of a small orally administered molecule that does not trigger an immune response and can cross the blood-brain barrier, potentially benefiting patients with neuronopathic forms of the disease. However, it is still unclear whether SSI impacts the prevention or reversal of neurological manifestations in patients. SSI has proven effective for visceral disease, similar to ERT, but hematological responses are slower and less effective compared to ERT. SSI’s side effects are more pronounced than ERT’s and may include diarrhea, tremors, paresthesia, headaches, arthralgia, weight loss, among others (Ceravolo et al., 2017; Mistry et al., 2017; Van Rossum and Holsopple, 2016; Wagner et al., 2018).

According to data from the Brazilian Ministry of Health, about 670 patients with GD are undergoing treatment in Brazil, with approximately 96% using ERT and 4% using SSI (Brasil, 2017). The ordinance no. 1,554 of 30 June 2013, regulating the Specialized Component of Pharmaceutical Assistance (CEAF), states that drug treatment should follow the lines of care defined in Clinical Protocols and Therapeutic Guidelines (PCDT), ensuring comprehensive care for patients, involving all evolutionary phases of the disease (Brasil, 2013). The Joint Ordinance No. 4 of 22 June 2017, establishes the current version of the PCDT for GD (Brasil, 2017).

GD presents high heterogeneity, thus analyzing the survival of these patients undergoing treatment in Brazil, understanding the main risk factors associated with survival, the profile of medication dispensation in the Brazilian National Health System (SUS), and the costs of those medications dispensed for GD treatment is extremely important to improve the management of these patients.

## 2 Methodology

### 2.1 Study design and population

The National Database from SUS in Brazil was used, developed through deterministic-probabilistic linkage technique using the databases of the Subsystem for Authorization of High-Cost/Complexity Procedures (APAC) from the Ambulatory Information System of SUS (SIA/SUS), which contains data on national outpatient care production, Hospital Admission Authorization (AIH) from the Hospital Information System of SUS (SIH/SUS), with data on national hospital care production, and Death Certificates (DO) from the Mortality Information System (SIM) with population-based mortality information. The methodological details of creating the National Database of SUS, using deterministic-probabilistic linkage, were described by Guerra and colleagues (2018) (Junior et al., 2018).

The study design is an open, non-concurrent cohort of all patients who were diagnosed according to ICD-10 as - E75.2 Other sphingolipidoses – Gaucher’s disease and who received ERT treatments imiglucerase, alfavelaglicerase, taliglucerase Alfa, and SSI miglustat, during the period from 2000 to 2015 in Brazil. The cohort entry date corresponded to the date of the first record of ERT or SSI dispensation. The entry period was from January 2000 to October 2015, and patients were followed from January 2000 to December 2015, totaling 16 years. Patients with a follow-up time of less than 3 months were excluded. Patients were censored if they abandoned or interrupted treatment for more than 3 months or at the end of the follow-up (right censorship).

To broaden the scope of our study, we incorporated data from TABNET system, managed by Department of Informatics of the Brazilian Unified Health System (DATASUS), which archives comprehensive medication distribution records. This extension allowed us to analyze the full spectrum of Gaucher disease medications dispensed across Brazil from 1999 to 2022. By leveraging TABNET, our objective was to scrutinize the volume of distributed medications, thereby gaining insights into treatment patterns that extend well beyond the initial cohort’s timeframe. This approach not only enriched our understanding of medication trends but also provided a quantitative baseline for future evaluations of pharmaceutical logistics and healthcare strategies for Gaucher disease nationwide.

### 2.2 Variables and statistical analysis

The primary event of interest for survival analysis was death. All patients were followed from the initial date until death or until December 2015 (right censoring), with loss of follow-up defined as informative censoring.

Baseline characteristics were described in a descriptive analysis of all variables based on the data recorded on the initial date. Explanatory variables included the sociodemographic characteristics of patients at the begin of the study. Weight and height information at baseline was used to calculate the body mass index (BMI) according to WHO parameters. Other variables included the Defined Daily Dose (DDD), medications, comorbidities, and region of residence (Liu et al., 2021).

Comorbidity scores were calculated based on the Charlson Comorbidity Index (CCI), considered a measure of patient severity, using medical service records in the database from the 3 years preceding the index date. Higher CCI indicates greater patient severity; low severity corresponds to a CCI between 0 and 1, and high severity to a CCI ≥2. The overall frailty of the patient (frailty index) was calculated as the number of days of hospitalization for any cause during the 2 years preceding the index date (Charlson et al., 1987; Quan et al., 2005).

Survival was assessed using the Kaplan-Meier method, and the log-rank test was used to compare the baseline characteristics and therapeutic regimens of patients. Factors influencing survival rates were initially evaluated by univariate analysis. Clinically relevant variables previously demonstrated in the literature and those with a *p*-value of <0.20 in univariate analysis were included in the multivariate Cox proportional hazards model. Adjusted hazard ratios (HRs) and 95% confidence intervals (CIs) were calculated in the multivariate model, and its suitability was assessed by residual analysis. Schoenfeld residuals were used to verify the proportional hazards assumption (SCHOENFELD, 1982).

The cost analysis approach centered on direct medical expenses from the Brazilian Ministry of Health’s records, considering only the SIA/SUS and SIH/SUS systems for average annual and overall expenditures per patient. This encompassed the costs for medications, outpatient, and hospital services annually. To ascertain the mean yearly expense per patient, we aggregated these individual costs and then calculated the central tendency for various categories such as gender, age, region, self-declared skin color, and specific GD medications. Additionally, we compiled data on prevalent comorbidities and complications to provide a comprehensive financial overview. The monetary values were adjusted according to the purchasing power parity index (PPP) of The World Bank (2024).

Statistical analysis was performed using R version 4.2.2 of the R Foundation for Statistical Computing.

### 2.3 Ethical considerations

The research was approved by the Research Ethics Committee of the Federal University of Minas Gerais (Opinion No. 16334413.9.0000.5149), ensuring that all patient data remained anonymous.

## 3 Results

### 3.1 Sample characteristics

**FIGURE 1:**
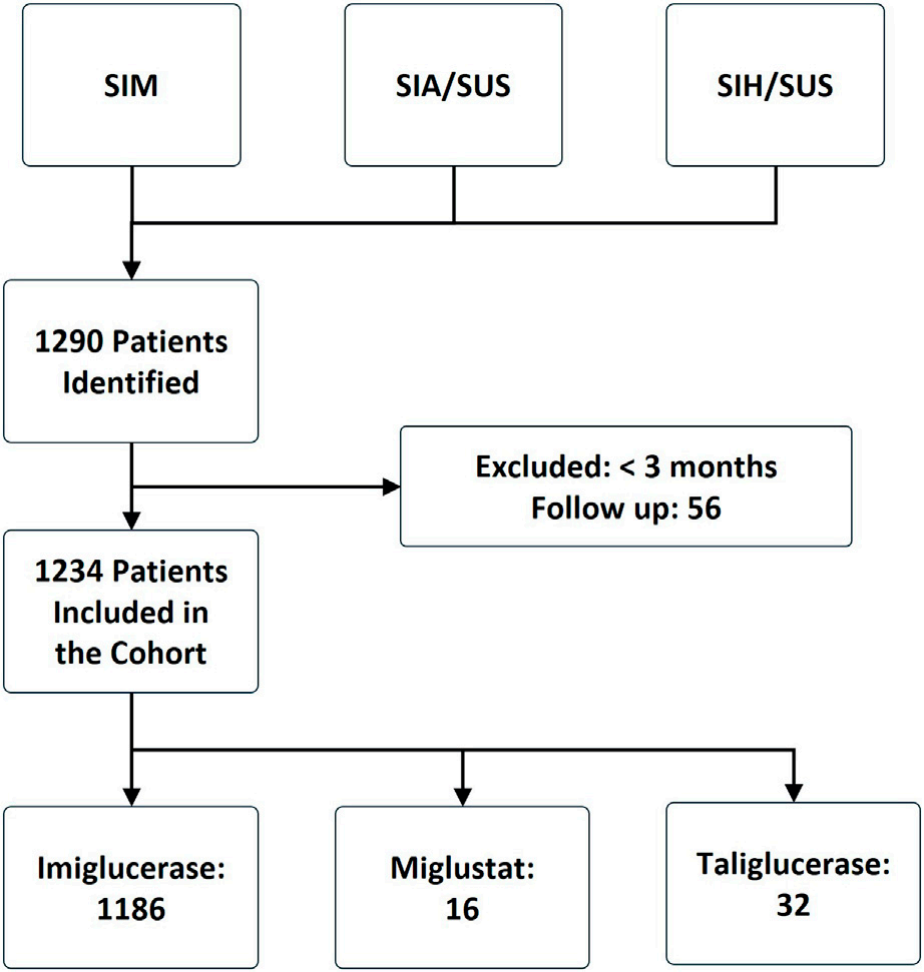
Flowchart of the construction of the Gaucher’s disease cohort, Brazil 2000–2015. SIA/SUS (ambulatory information system of SUS), SIH/SUS (hospital information system of SUS), and SIM (mortality information system).

From January 2000 to December 2015, 1186 (96%) patients used imiglucerase, 16 (1.3%) used miglustat, and 32 (2.6%) used taliglucerase Alfa (Figure 1). The median age of patients in the cohort was 22 years, with a median age of 21 years in the imiglucerase group, 40 years in the miglustat group, and 22 years in the taliglucerase alfa group. The majority of patients were from the Southeast region (57%), followed by the Northeast region (19%). From 2000 to 2007, patients entered the cohort receiving only imiglucerase. From 2008 to 2011, entries for taliglucerase alfa were recorded, while entries for miglustat were only recorded from 2012 to 2015. The characteristics of the study population are summarized in Table 1.

**TABLE 1:** Baseline characteristics of patients included in the cohort.

Characteristic		Imiglucerase, N = 1,186	Miglustat, N = 16	Taligucerase alfa, N = 32	Full cohort, N = 1234
Sex	Female	703 (59%)	14 (88%)	18 (56%)	735 (60%)
	Male	483 (41%)	2 (12%)	14 (44%)	499 (40%)
Age at baseline		21 (11, 37)	40 (28, 43)	36 (23, 57)	22 (11, 38)
Age range at baseline	>65 years	25 (2.1%)	1 (6.2%)	1 (3.1%)	27 (2.1%)
	0–11 years	331 (28%)	0 (0%)	2 (6.2%)	333 (27%)
	12–17 years	182 (15%)	0 (0%)	5 (16%)	187 (15%)
	18–25 years	166 (14%)	2 (12%)	2 (6.2%)	170 (14%)
	26–35 years	185 (16%)	3 (19%)	6 (19%)	194 (16%)
	36–45 years	132 (11%)	9 (56%)	4 (12%)	145 (12%)
	46–55 years	121 (10%)	1 (6.2%)	4 (12%)	126 (10%)
	56–65 years	44 (3.7%)	0 (0%)	8 (25%)	52 (4.1%)
Self-declared skin color	Yellow (Asian)	69 (5.8%)	3 (19%)	4 (12%)	76 (6.2%)
	White	119 (10%)	0 (0%)	2 (6.2%)	121 (9.8%)
	Unknown	932 (79%)	13 (81%)	23 (72%)	968 (78%)
	Brown	47 (4.0%)	0 (0%)	2 (6.2%)	49 (4.0%)
	Black	19 (1.6%)	0 (0%)	1 (3.1%)	20 (1.6%)
Residence region at baseline	Midwest	69 (5.8%)	3 (19%)	4 (12%)	76 (6.2%)
	Northeast	234 (20%)	1 (6.2%)	2 (6.2%)	237 (19%)
	North	57 (4.8%)	4 (25%)	0 (0%)	61 (4.9%)
	Southeast	672 (57%)	4 (25%)	25 (78%)	701 (57%)
	South	154 (13%)	4 (25%)	1 (3.1%)	159 (13%)
Cohort entry period	2000 a 2003	431 (36%)	0 (0%)	0 (0%)	431 (35%)
	2004 a 2007	250 (21%)	0 (0%)	0 (0%)	250 (20%)
	2008 a 2011	351 (30%)	0 (0%)	27 (84%)	378 (31%)
	2012 a 2015	154 (13%)	16 (100%)	5 (16%)	175 (14%)
Body mass index at baseline	Normal weight	47 (4.0%)	0 (0%)	3 (9.4%)	50 (4.1%)
	Obesity	236 (20%)	12 (75%)	24 (75%)	272 (22%)
	Overweight	36 (3.0%)	3 (19%)	3 (9.4%)	42 (3.4%)
	Severely underweight	28 (2.4%)	0 (0%)	0 (0%)	28 (2.3%)
	Underweight	16 (1.3%)	0 (0%)	0 (0%)	16 (1.3%)
	Unknown	823 (69%)	1 (6.2%)	2 (6.2%)	826 (67%)
Dose	Equal to DDD	15 (1.3%)	0 (0%)	0 (0%)	15 (1.2%)
	Higher than DDD	325 (27%)	14 (88%)	0 (0%)	339 (27%)
	Lower than DDD	846 (71%)	2 (12%)	32 (100%)	880 (71%)
Comorbidities	Diabetes	7 (0.6%)	0 (0%)	1 (3.1%)	8 (0.6%)
	Cardiovascular Disease	15 (1.3%)	0 (0%)	2 (6.2%)	17 (1.4%)
	Parkinson Disease	10 (0.8%)	0 (0%)	0 (0%)	10 (0.8%)
	Cancer	10 (0.8%)	0 (0%)	2 (6.2%)	12 (1.0%)
Complications	Splenectomy	14 (1.2%)	0 (0%)	1 (3.1%)	15 (1.2%)
	Splenomegaly	35 (3.0%)	0 (0%)	3 (9.4%)	38 (3.1%)
	Hepatomegaly	30 (2.5%)	0 (0%)	1 (3.1%)	31 (2.5%)
	Anemia	1,053 (89%)	16 (100%)	32 (100%)	1,101 (89%)
	Thrombocytopenia	10 (0.8%)	1 (6.2%)	0 (0%)	11 (0.9%)
	Bone and Muscle Events	109 (9.2%)	3 (19%)	4 (12%)	116 (9.4%)
	Infections	27 (2.3%)	0 (0%)	4 (12%)	31 (2.5%)
Frailty index		10 (4, 21)	15 (15, 15)	18 (4, 40)	10 (4, 22)
Event Type	Censoring	1,124 (95%)	16 (100%)	30 (94%)	1,170 (95%)
	Death	62 (5.2%)	0 (0%)	2 (6.2%)	64 (5.2%)

### 3.2 Survival analysis

**FIGURE 2:**
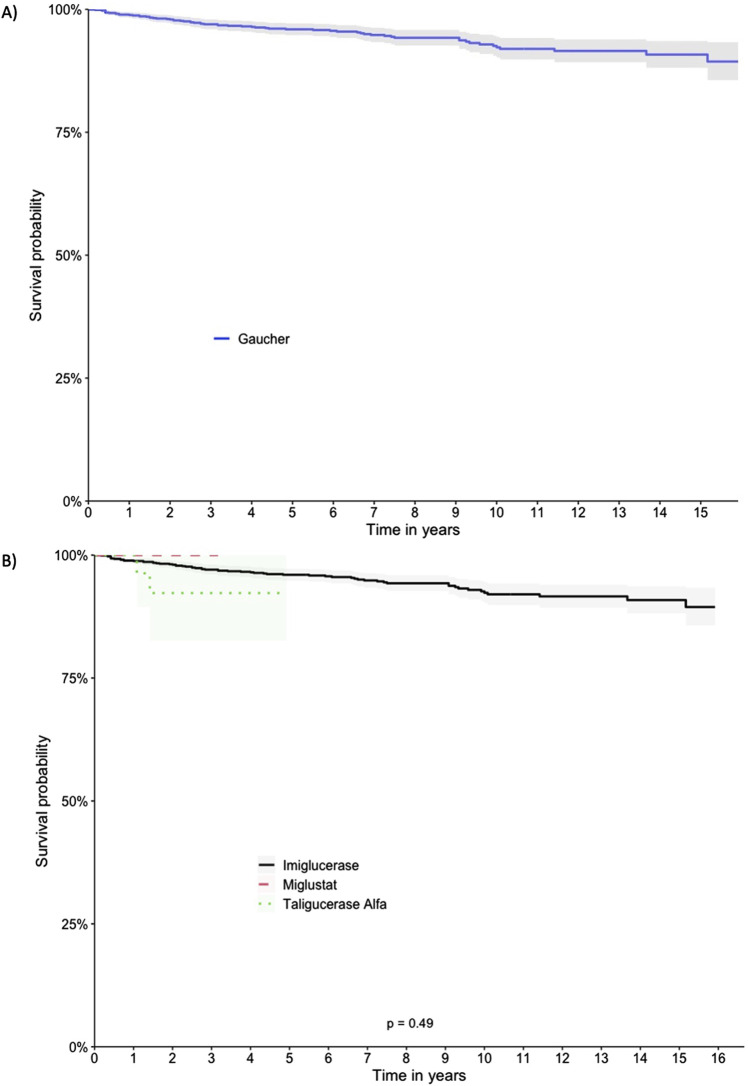
Kaplan-Meier Survival Curves for the Full Cohort and Comparing Therapies. **(A)** Full cohort, **(B)** Therapies.

**FIGURE 3:**
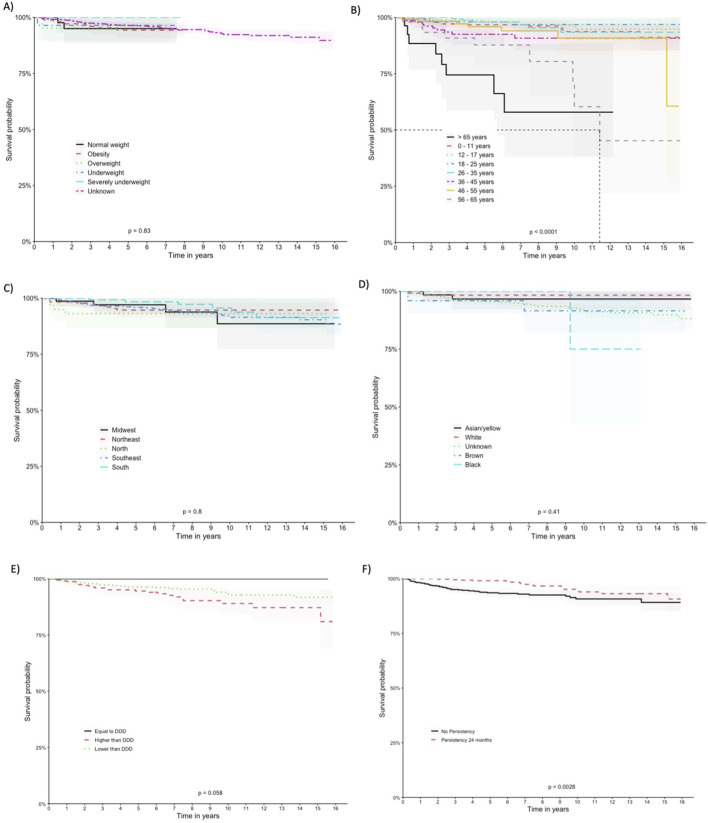
Kaplan-Meier Curves According to Patient Characteristics. **(A)** BMI, **(B)** age at entry, **(C)** region of residence, **(D)** self-declared race/color, **(E)** Defined Daily Dose, and **(F)** drug-survival.

**FIGURE 4:**
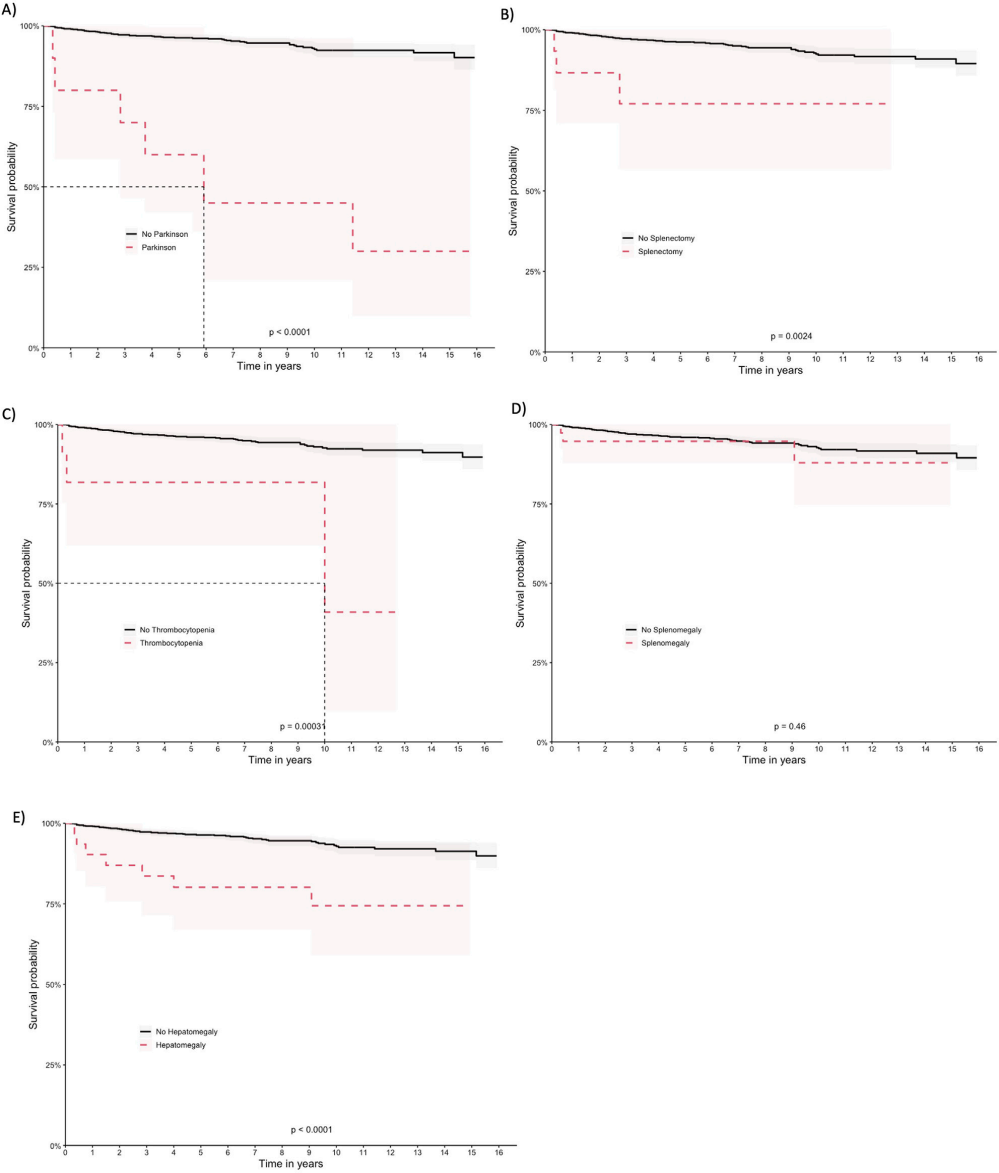
Kaplan-Meier curves for comorbidities and complications **(A)** Parkinson disease, **(B)** splenectomy, **(C)** thrombocytopenia, **(D)** splenomegaly, **(E)** hepatomegaly.

The patients exhibited survival rates of 98.8%, 95.9%, 92.3%, and 89.4% at one, five, ten, and 15 years respectively. A total of 64 deaths occurred in the cohort (5.2%), with 62 in the imiglucerase group and 2 in the taliglucerase alfa group. No deaths were identified in the miglustat group. Figure 2 displays the survival curve for the entire cohort, as well as the survival curve comparing the therapies received. Figures 3, 4 show the survival curves according to patient characteristics. Patients aged 56–65 years, with thrombocytopenia, hepatomegaly, splenomegaly, who underwent splenectomy, and those diagnosed with Parkinson’s Disease have significantly lower survival than the rest of the cohort.

We observed variations in survival probabilities based on the adherence to the DDD of imiglucerase. Notably, patients who were administered doses below the recommended DDD, comprising the largest subgroup (n = 880), demonstrated a survival probability of 91.8%. Contrastingly, the group adhering precisely to the DDD (n = 15), although showing a 100% survival probability, represents a significantly smaller sample size, necessitating cautious interpretation of this perfect survival rate. Patients receiving above the recommended DDD (n = 339) showed a survival probability of 81%. The statistical analysis, including a log-rank test, indicated borderline significance (*p* = 0.058), with the lower dose cohort displaying a trend towards better survival outcomes.

### 3.3 Univariate analysis

The univariate analysis detailed in Table 2 indicates that age was a significant factor for survival in patients with Gaucher’s disease. Patients aged 0–11 years (HR 0.10 [95% CI: 0.04–0.23]), 12–17 years (HR 0.06 [95% CI: 0.02–0.19]), 18–25 years (HR 0.05 [95% CI: 0.02–0.18]), and 26–35 years (HR 0.07 [95% CI: 0.03–0.21]) had a lower risk of death compared to the reference group of patients over 65 years. Regarding sex, there was no significant difference in survival rates between male and female patients. Additionally, the patients’ region of residence at the start of the study was not a significant factor for survival. However, the presence of comorbidities such as diabetes (HR 26.4 [95% CI: 10.4–66.9]), cardiovascular disease (HR 6.12 [95% CI: 1.91–19.7]), Parkinson’s disease (HR 13.5 [95% CI: 5.81–31.3]), splenectomy (HR 5.06 [95% CI: 1.59–16.1]), hepatomegaly (HR 4.42 [95% CI: 2.01–9.70]), infections (HR 5.64 [95% CI: 2.42–13.1]), and cancer (HR 15.4 [95% CI: 6.15–38.7]) were all associated with an increased risk of death.

**TABLE 2:** Univariate analysis.

Characteristic		HR^a^	95% CI^a^	*p*-value
Sex	Female	1.00	—	
	Male	1.38	0.84, 2.25	0.2
Age range at baseline	>65 years	1.00	—	
	0–11 years	0.10	0.04, 0.23	**<0.001**
	12–17 years	0.06	0.02, 0.19	**<0.001**
	18–25 years	0.05	0.02, 0.18	**<0.001**
	26–35 years	0.07	0.03, 0.21	**<0.001**
	36–45 years	0.19	0.08, 0.49	**<0.001**
	46–55 years	0.15	0.05, 0.42	**<0.001**
	56–65 years	0.51	0.20, 1.33	**0.2**
Self-declared skin color	Yellow (asian)	1.00	—	—
	White	0.54	0.08, 3.86	0.5
	Unknown	1.85	0.45, 7.58	0.4
	Brown	2.03	0.34, 12.2	0.4
	Black	1.91	0.17, 21.1	0.6
Residence region at baseline	Midwest	1.00	—	
	Northeast	0.94	0.29, 2.99	>0.9
	North	1.28	0.32, 5.13	0.7
	Southeast	0.98	0.35, 2.75	>0.9
	South	0.61	0.17, 2.15	0.4
Cohort entry period	2000 a 2003	1.00	—	
	2004 a 2007	1.79	0.96, 3.33	**0.067**
	2008 a 2011	1.10	0.53, 2.31	0.8
	2012 a 2015	3.39	1.40, 8.18	**0.007**
Body mass index at baseline	Normal weight	1.00	—	
	Obesity	1.02	0.23, 4.61	>0.9
	Overweight	1.14	0.16, 8.08	0.9
	Severely underweight	0.84	0.08, 9.25	0.9
	Underweight	0.00	0.00, Inf	>0.9
	Unknown	0.70	0.17, 2.92	0.6
Dose	Equal to DDD	1.00	—	
	Higher than DDD	13,856,501	0.00, Inf	>0.9
	Lower than DDD	7,976,042	0.00, Inf	>0.9
Presence of Comorbidities	Diabetes	26.4	10.4, 66.9	**<0.001**
	Cardiovascular Disease	6.12	1.91, 19.7	**0.002**
	Parkinson Disease	13.5	5.81, 31.3	**<0.001**
	Cancer	15.4	6.15, 38.7	**<0.001**
Presence of Complications	Splenectomy	5.06	1.59, 16.1	**0.006**
	Splenomegaly	1.55	0.49, 4.94	0.5
	Hepatomegaly	4.42	2.01, 9.70	**<0.001**
	Anemia	1.68	0.75, 3.76	0.2
	Thrombocytopenia	6.42	2.01, 20.5	**0.002**
	Bone and Muscle Events	1.45	0.74, 2.85	0.3
	Infections	5.64	2.42, 13.1	**<0.001**
Frailty index				
Charlson Comorbidity Index		1.10	1.06, 1.14	**<0.001**
Medicine	Imiglucerase	1.00	—	
	Miglustat	0.00	0.00, Inf	>0.9
	Taligucerase Alfa	2.12	0.51, 8.77	0.3

^a^ HR, hazard ratio; CI, confidence interval.

Bold values indicate statistical significance.

### 3.4 Multivariate analysis

Considering the statistical significance level used in the univariate analysis (*p* < 0.20) and relevant epidemiological clinical data, multivariate analyses were performed (Table 3). Regarding medications, the use of miglustat did not have a significant effect on survival compared to imiglucerase, while taliglucerase alfa had an HR of 1.65 (95% CI 0.35, 7.83), although not statistically significant. Among the comorbidities studied, Parkinson’s disease presented the highest risk (HR 12.2 [95% CI: 4.85, 30.6]), followed by hepatomegaly (HR 5.67 [95% CI: 2.15, 15.0]) and thrombocytopenia (HR 5.92 [95% CI: 1.77, 19.8]). In contrast, splenomegaly had a protective effect (HR 0.17 [95% CI: 0.03, 0.90]), indicating a lower risk of death. The correction for false discovery rate (FDR) in multivariate analysis was performed, and some associations remained statistically significant, such as Parkinson’s disease, splenectomy, hepatomegaly, and thrombocytopenia. The Schoenfeld residual analysis demonstrated that the multivariate model had good fit, with an average close to zero and without violation of the homoscedasticity assumption.

**TABLE 3:** Multivariate analysis.

Characteristic		HR^a^	95% CI^a^	*p*-value	q-value^b^
Medicine	Imiglucerase	—	—		
	Miglustat	0.00	0.00, Inf	>0.9	>0.9
	Taligucerase Alfa	1.65	0.35, 7.83	0.5	0.7
Comorbidities/Complications	Parkinson Disease	12.2	4.85, 30.6	<0.001	**<0.001**
	Splenectomy	7.28	1.58, 33.6	0.011	**0.025**
	Splenomegaly	0.17	0.03, 0.90	0.037	0.067
	Hepatomegaly	5.67	2.15, 15.0	<0.001	**0.002**
	Thrombocytopenia	5.92	1.77, 19.8	0.004	**0.011**
	Bone and Muscle Events	1.07	0.52, 2.21	0.9	>0.9

^a^ HR, hazard ratio; CI, confidence interval.

^b^ False discovery rate correction for multiple testing.

Bold values indicate statistical significance.

### 3.5 Cost analysis

Tables 4 detail the overall medical care annual spending, revealing significant expenditures on imiglucerase, miglustat, and taliglucerase alfa. The data encapsulates a comprehensive financial overview, considering factors such as patient demographics, weight categories, and regional distribution of expenses. Notably, imiglucerase represented the most substantial portion of the total cost, reflecting its predominance in GD management. The introduction of alternative treatments like miglustat and taliglucerase alfa also contributed to overall expenses, albeit to a lesser extent.

**TABLE 4:** Mean annual overall medical care costs in USD PPP (SIA/SIH) by Baseline Characteristics of Patients Included in the 2000–2015 Cohort.

Characteristic		Imiglucerase (USD PPP)	Miglustat (USD PPP)	Taligucerase alfa (USD PPP)	Full cohort (USD PPP)
Sex	Female	124.603.61	27.589.45	48.017.12	120.880.14
	Male	121.894.66	41.419.19	75.284.40	120.264.41
Age range at baseline	>65 years	141.728.22	41.007.19	22.280.82	133.573.83
	0–11 years	96.755.89		33.974.24	96.378.82
	12–17 years	129.419.92		50.102.76	127.299.14
	18–25 years	130.414.07	44.875.91	35.272.62	128.288.43
	26–35 years	132.400.30	8.216.05	60.002.94	128.240.83
	36–45 years	138.979.84	32.055.43	90.225.49	130.998.21
	46–55 years	145.509.57	25.184.56	55.956.60	141.711.66
	56–65 years	119.382.76		70.281.92	111.828.78
Self-declared skin color	Yellow (asian)	88.406.29	14.140.52	66.450.11	84.319.15
	White	111.876.32		91.090.42	111.532.75
	Unknown	128.879.21	32.820.70	52.421.96	125.772.52
	Brown	106.652.09		86.451.85	105.827.59
	Black	101.582.89		91.699.66	101.088.73
BMI	Normal weight	68.122.73		67.219.97	68.068.57
	Obesity	85.975.03	24.792.84	60.638.79	81.040.27
	Overweight	62.316.87	34.807.57	74.341.74	61.210.84
	Severely underweight	43.288.16			43.288.16
	Underweight	51.668.04			51.668.04
	Unknown	144.225.30	67.153.87	19.136.83	143.829.11
Residence region at baseline	Midwest	117.093.86	33.679.92	52.287.34	110.390.34
	Northeast	100.404.82	0.00	85.315.79	99.853.84
	North	81.141.23	17.625.48		76.976.26
	Southeast	140.741.59	39.648.36	60.490.35	137.302.71
	South	101.908.18	34.738.89	26.249.87	99.742.55
Dose	Equal to DDD	158.298.51			158.298.51
	Higher than DDD	163.586.51	33.428.84		158.211.27
	Lower than DDD	107.483.88	543.41	59.946.55	105.512.21
Comorbidities	Diabetes	167.977.90		18.395.66	149.280.12
	Cardiovascular Disease	128.620.44		17.194.25	115.511.48
	Parkinson Disease	166.446.39			166.446.39
	Cancer	130.537.53		15.892.17	111.429.97
Complications	Splenectomy	149.310.42		154.638.73	149.665.64
	Splenomegaly	124.403.59		109.180.81	123.201.79
	Hepatomegaly	138.459.27		57.117.98	135.835.36
	Anemia	115.065.88	29.318.16	59.946.55	112.217.76
	Thrombocytopenia	127.244.92	67.153.87		121.782.09
	Bone and Muscle Events	152.361.44	23.138.36	71.294.70	146.224.06
	Infections	104.021.06		40.366.50	95.807.57

### 3.6 Drug distribution analysis

**FIGURE 5:**
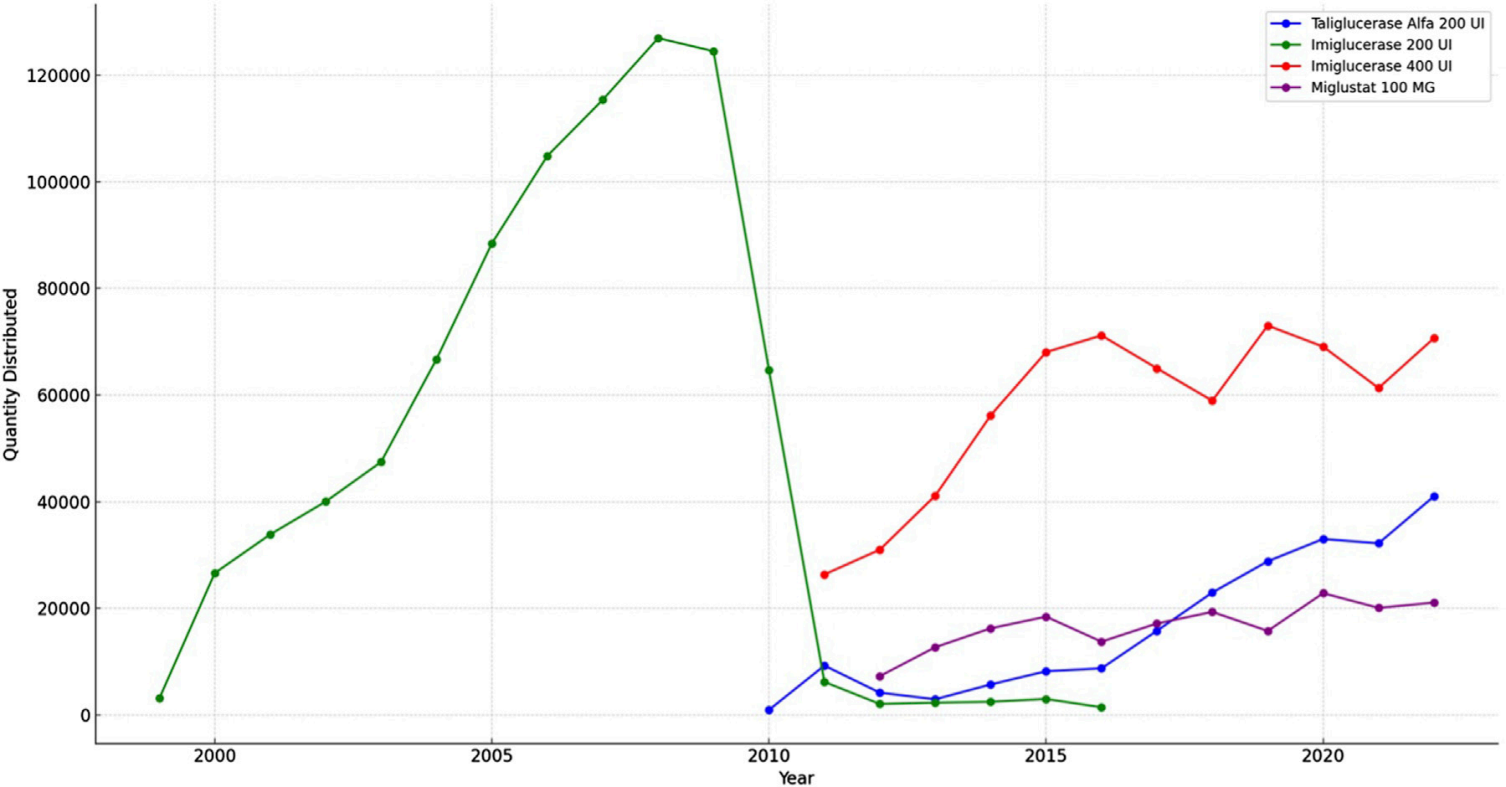
Trends in Gaucher disease medicines distribution (1999–2022).

The results from the TABNET system data, depicted in Figure 5 indicate that the distribution of imiglucerase 200 U, quantified in units delivered to patients, escalated until 2008 before experiencing a gradual decline. Concurrently, the dispensation of miglustat 100 mg showed relative consistency throughout the observed years. The alfataliglicerase 200 U, while introduced later within the study period, demonstrated a steady uptake. This graphical representation of medication distribution provides a clear visualization of the longitudinal dispensation patterns for Gaucher disease treatments in Brazil.

## 4 Discussion

This study provides a comprehensive analysis of Gaucher disease (GD) in Brazil, focusing on survival rates, treatment patterns, and associated costs over a 16-year period. By leveraging data from multiple national health databases, we offer valuable insights into the management of GD within the Brazilian Unified Health System (SUS), one of the world’s largest public healthcare systems. Our findings contribute to the growing body of knowledge on GD, particularly from a non-Western perspective, and highlight the unique challenges and successes in treating this rare disease in Brazil.

The apparent discrepancy between the number of treated patients cited in previous studies and the cohort size in our study can be explained by the broader inclusion criteria used in our analysis. While previous reports, such as those published in 2017, focused on the number of patients actively undergoing treatment at a specific point in time, our study includes all patients identified with Gaucher disease in the Brazilian Unified Health System (SUS) databases from 2000 to 2015. This comprehensive approach allowed us to capture a wider spectrum of the Gaucher disease population, including those who may have been diagnosed but were not actively receiving treatment at the time of the cited studies. Furthermore, our cohort reflects the entire study period, which provides a more robust understanding of the disease’s prevalence and treatment patterns in Brazil over 16 years. This difference in study design underscores the importance of considering both cross-sectional and longitudinal data when evaluating patient populations in rare diseases like Gaucher disease (Mistry et al., 2017).

The findings from the cost analysis highlight the significant financial investment by the Brazilian Ministry of Health in managing Gaucher’s disease. The data emphasizes the economic impact of chronic disease management and the importance of cost-effective therapeutic strategies. These insights are vital for policy-making and allocation of resources, ensuring sustainable patient care within the public health system.

The survival analysis results align with other studies assessing imiglucerase’s effectiveness in Gaucher’s disease patients. Weinreb and Lee (2013) reported that long-term imiglucerase use was associated with significant pain score reduction, quality of life improvements, increased body weight, and better hematological parameters. However, patient survival was not an outcome evaluated in their study. It's crucial to note that Gaucher’s disease is rare and complex, with varied clinical manifestations, which may complicate comparisons across different studies.

Consistent with previous reports of clinical improvements, such as those detailed by Tukan et al. (2013), our data suggest that these benefits may extend to patient survival, particularly when considering dosage adherence. Although the perfect survival rate within the DDD group is encouraging, the small sample size limits the generalizability of this finding. In contrast, the lower dose group’s favorable survival trend, with its substantially larger patient population, suggests that reduced imiglucerase dosing could be associated with improved survival outcomes. These observations advocate for personalized dosing strategies that account for individual patient needs and responses to treatment, aligning with the single-center experience reported by Tukan et al. (2013) which highlighted the achievement of therapeutic goals with low-dose imiglucerase. It is imperative to conduct further research with a larger sample size for each dosing category to elucidate the optimal imiglucerase dosing strategy for enhancing survival in Gaucher disease while minimizing potential adverse effects.

Miglustat therapy has also been evaluated in patients with Gaucher’s disease. Kuter et al. (2013) found that miglustat was associated with a significant reduction in splenomegaly, an increase in platelet count, and a decrease in the number of blood transfusions required. Furthermore, miglustat treatment was well tolerated by patients, with no serious adverse reactions observed. However, this retrospective study did not assess patient survival rates (Kuter et al., 2013).

An important aspect of our study is the analysis of treatment trends over time, particularly the introduction of newer therapies. Eliglustat, a substrate reduction therapy, was registered in Brazil during our study period but was not prescribed within the SUS up to 2022. The lack of eliglustat use in the public healthcare system reflects broader challenges in integrating new treatments into public health formularies, especially in countries with resource constraints. This finding underscores the need for ongoing policy discussions and strategic planning to ensure that patients with rare diseases like GD have access to the most effective and up-to-date treatments available (Iriart et al., 2019).

Regarding the clinical characteristics of patients, the survival analysis revealed that patients with thrombocytopenia, hepatomegaly, splenomegaly, those who underwent splenectomy, and those diagnosed with Parkinson’s disease had significantly lower survival rates than the rest of the cohort. These findings are consistent with the results reported by El-Beshlawy et al. (2017) in children with Gaucher type 3 disease treated with imiglucerase. This study showed that imiglucerase treatment was associated with a significant improvement in hematological parameters, a reduction in splenomegaly and hepatomegaly, as well as a significant improvement in the height and weight of patients (El-Beshlawy et al., 2017).

Comparing the results obtained in this retrospective cohort with other populations, the study by Jaffe et al. (2019) describing a cohort of 500 patients with Gaucher Disease in Israel stands out. Although there are differences in the sample and methodology between the studies, the survival rate observed in the present cohort was similar to that described in the Israeli study, indicating that treatment for Gaucher Disease can be effective in different contexts and populations. However, it is important to note that the sample of this cohort mainly consisted of patients who received imiglucerase, while in Israel, taliglucerase alfa is the first-line therapy. This difference may have an impact on survival outcomes and should be considered when comparing results between the studies (Jaffe et al., 2019).

Furthermore, the systematic review and meta-analysis by Leonart et al. (2023) provide a comprehensive overview of the available evidence on treatments for Gaucher Disease. The authors highlight that while imiglucerase is the most common therapy, there is evidence that other treatments, such as taliglucerase alfa and Miglustat, are also effective. The results of this study, which included patients who received these therapies, support these findings and suggest that these alternative treatments may be a viable option for patients with Gaucher Disease (Leonart et al., 2023).

The findings of this study indicate that the age at the start of treatment with imiglucerase was significantly lower in patients with more severe forms of Gaucher Disease, as evidenced by a higher proportion of patients with prior splenectomy in this group. These findings suggest that early identification of Gaucher Disease may be important in preventing or minimizing disease progression and the occurrence of complications. The development of sensitive and specific biomarkers, such as glucosylsphingosine, may help improve the early identification and monitoring of patients with Gaucher Disease.

In terms of treatment, imiglucerase was the primary therapeutic agent used in this cohort of Brazilian patients with Gaucher Disease. This finding is in line with the current literature, which emphasizes the effectiveness and safety of imiglucerase in the treatment of Gaucher Disease. However, new therapies for Gaucher Disease are being developed, such as gene therapy and therapy with glucocerebrosidase inhibitors, which may provide additional therapeutic options for patients with Gaucher Disease (Jaffe et al., 2019; Koppe et al., 2016; Leonart et al., 2023).

Other complications and comorbidities in patients with Gaucher Disease have been extensively studied. The prevalence of neurological manifestations in patients with the non-neuronopathic form of Gaucher Disease was recently reviewed in a Dutch study, which identified that 23% of patients had neurological symptoms. However, larger and prospective population studies are still needed to better understand the prevalence of these comorbidities. Additionally, studying patients with Gaucher Disease and their comorbidities can help elucidate the underlying mechanisms of these conditions and improve treatment strategies (Lopez et al., 2020; Goker-Alpan and Ivanova, 2024).

Is noteworthy to mention the distribution trend for GD medicines in Brazil. In 2010, the global imiglucerase shortage, triggered by viral contamination at the manufacturing facility in the USA, posed a significant challenge for Gaucher’s disease management worldwide (O Globo, 2010). The Brazilian response, prioritizing patient care, involved the emergent use of taliglucerase alfa. This plant-derived recombinant enzyme was provisionally utilized before its formal approval in 2012, reflecting the urgency to circumvent the shortage (Formenti, 2010). The Ministry of Health’s updated PCDT guidelines in 2011, included miglustat, taliglucerase alfa, and velaglucerase alfa in the SUS treatment protocol for Gaucher’s disease. However, the adoption of velaglucerase alfa remained minimal, as indicated by the distribution data, suggesting potential regulatory, availability, or clinical practice barriers (Brasil, 2011).

The transition from 200 UI to 400 UI of imiglucerase, as confirmed in the ANVISA registration records, implies a strategic choice by the manufacturer, possibly aligning with global supply adjustments. This shift, while not directly addressed in the PCDT, has practical implications for dosing regimens in Brazil. These strategic responses highlight the adaptability of the Brazilian healthcare system to global pharmaceutical events and underscore the importance of flexible national health policies in ensuring uninterrupted patient treatment (ANVISA, 2024).

The Brazilian population’s ethnic and genetic diversity presents both challenges and opportunities in understanding GD’s natural history and treatment outcomes. While our study included variables such as skin color and region of residence, the high number of unknowns in some categories, particularly skin color, limits the depth of our analysis. Nevertheless, these variables are crucial for exploring potential health disparities and understanding how different population subgroups respond to GD treatment. The inclusion of these variables aligns with our study’s goal of providing a more detailed and contextually relevant analysis of GD in Brazil (Souza et al., 2019).

The main limitation of this study is that the data collected from the administrative systems developed by the Brazilian Unified Health System (SUS) were not designed to assess and track clinical outcomes. Consequently, there is no record of patient-level clinical variables, such as hemoglobin concentration, platelets, spleen, and liver volume. Our analysis assumes that the distribution of medications equates to their administration. However, this is an indirect measure and may not perfectly reflect actual patient exposure to the medications. Additionally, there is the possibility of incorrect data due to underreporting, which can lead to underestimation or overestimation of the analyses conducted. It is likely that some events were not recorded in the SUS databases, as many patients are known to use private healthcare services and only rely on SUS for medication procurement. Another limitation relates to the quality of the data used, which depends on the process of recording and inputting secondary information into the original database. Incorrect or incomplete data - an inherent limitation of secondary databases - can underestimate or overestimate the results of the analysis, especially for clinical variables and non-mandatory fields such as weight and race.

Despite these limitations, we believe that our results are robust enough to contribute to the data on Gaucher disease treatment in low and middle-income countries and may provide evidence for future health policies.

## 5 Conclusion

The analysis of survival in patients undergoing treatment for Gaucher disease in the Brazilian public healthcare system has shown important results in terms of improving survival and identifying risk factors for mortality. The study contributes to the growing body of literature on Gaucher disease, highlighting the importance of early diagnosis, timely initiation of treatment, monitoring and management of risk factors, and personalized dosing strategies. Future research should focus on further elucidating the genetic and molecular mechanisms underlying Gaucher disease and its association with other conditions, as well as identifying new biomarkers and therapeutic targets for personalized treatment approaches.

## Data Availability

The datasets presented in this article are not readily available because the administrative databases used were provided by the Ministry of Health to the University in order to carry out epidemiological and research to guide decision making in health and for this will not be publicized. . Requests to access the datasets should be directed to datasus@saude.gov.br.
